# Date Palm Fruit (*Phoenix dactylifera*) and Its Promising Potential in Developing Functional Energy Bars: Review of Chemical, Nutritional, Functional, and Sensory Attributes

**DOI:** 10.3390/nu15092134

**Published:** 2023-04-28

**Authors:** Hassan Barakat, Hani A. Alfheeaid

**Affiliations:** 1 Department of Food Science and Human Nutrition, College of Agriculture and Veterinary Medicine, Qassim University, Buraydah 51452, Saudi Arabia; h.alfheeaid@qu.edu.sa; 2 Food Technology Department, Faculty of Agriculture, Benha University, Moshtohor 13736, Egypt; 3 School of Medicine, Dentistry and Nursing, College of Medical, Veterinary and Life Sciences, University of Glasgow, Glasgow G12 8QQ, UK

**Keywords:** *Phoenix dactylifera*, energy bars, date bars, functional foods, food security, nutrition

## Abstract

Snack bars, known as energy bars, are widely consumed worldwide as highly nutritive on-the-go products. Due to the date fruit’s significant nutritional and functional characteristics, it can be an exceptional choice for developing snack bars. Dates contain a wide range of macro- and micronutrients known for their strong bioactive properties. The functional properties of date fruit have been demonstrated in the literature and include antioxidant, anti-inflammatory, anti-tumor, antihypertensive, and antimicrobial activities. This review summarizes the available studies investigating the potential application of dates for developing nutritive and functional snack bars. Date paste was used as a main ingredient at 55–90% concentrations. In addition, protein sources were used to provide protein-rich snack bars, as date fruit is considered high in carbohydrates and low in protein. Skim milk powder was the most common and favorable protein source, delivering significant amounts of protein with limited negative effects on sensory attributes. Incorporating other ingredients, such as cereals or legumes, was also explored; adding such dry ingredients can promote positive nutritional effects along with improving sensory attributes, mainly in terms of the bars’ textures. Dry ingredients can significantly lower moisture content, reducing the bars’ fracturability to acceptable ranges. Reduced moisture content can also significantly enhance the shelf-life stability, as observed by limited microbial growth. Furthermore, the incorporation of bioactive or functional ingredients such as fruit peels, plant seeds, or plant leaf extracts was also reported; such ingredients promoted significant enhancements in the contents of phenolics or flavonoids, for instance, leading to an increase in the bars’ antioxidant potential. Though dates are rich in such bioactive components, incorporating additional bioactive ingredients can boost the dates’ functional properties. In conclusion, this review shows the high potential of the application of dates for developing nutritious and functional snack bars. Taking this into account, the snack bar market has grown remarkably over the past decade; thus, providing well-balanced, nutritious, and functional date-based bars in markets worldwide is expected to show positive consumer acceptance.

## 1. Introduction

The demand for easy and immediate nutrient-dense meal replacement has been growing for the past two decades with growing concerns about health and fitness. Commercial bar-shaped snack products, known as snack or energy bars, are an excellent choice and the most common on-the-go products serving consumers’ health demands. Snack bars are mostly manufactured to provide good amounts of nutrients and serve as energy boosters, thus gaining the common term “energy bars”. Snack bars can be enjoyable, suitable, and equally nutritious for all age groups but are mostly consumed by highly active individuals such as athletes [1,2]. The global snack bar market has grown remarkably over the past decade; it is expected to increase from USD 15 billion in 2019 to 19 billion by 2025 [3]. Several hundred types of on-the-go packed snack bars are present in the market nowadays; balanced, protein-enriched, cereal breakfast replacement, and brain-boosting bars are among the available types. Snack bars mostly consist of different food sources, such as cereals, legumes, fruits, and nuts, with additional coatings or the incorporation of chocolate chips [4]. Fruit-based snack bars are among the most favorable, providing a highly nutritious product containing natural sugars, vitamins, minerals, and other bio-nutritive components that meet consumers’ required daily nutritional intake [1].

Due to its rich nutritional composition, the date palm fruit can be an exceptional choice for manufacturing snack bars. Although they are considered a rich source of carbohydrates, dates can provide significant amounts of good-quality nutrients, such as dietary fiber (e.g., β-glucans), unsaturated fatty acids (e.g., oleic and linoleic acids), and a wide range of micronutrients (e.g., riboflavin, niacin, tocopherols, potassium, and calcium). Dates are also considered low in protein, though studies have found significant essential amino acids in dates, such as lysine and histidine, that are lacking in most fruits. In addition, in dates, various types of bioactive phytochemicals, such as phenolic acids, polyphenols, and carotenoids, were found in significant amounts [5,6,7,8]. Such bioactive nutrients in any food source can significantly boost its potential functional properties [9]; dates’ bioactive components were significantly correlated in some studies with their antioxidant potential [10,11]. Some other studies reported significantly high antioxidant capacities in multiple date varieties, ranging from 55 to 75% [12]. Furthermore, recent in vivo and in vitro studies have reported different functional or pharmacological effects of date consumption. Dates were shown to promote health-beneficial effects such as anti-inflammatory, anti-tumor, antihypertensive, anti-hypercholesterolemia, and antimicrobial effects [13,14,15,16,17,18,19].

Apart from the substantial nutritional aspects of dates, from an economic point of view, dates are considered highly valuable. Their cultivation has shown a significant growth trend over the past three decades. Date production is widespread worldwide, mainly in Middle Eastern and Northern African countries, which share more than 70% of world production. Countries such as Egypt and Saudi Arabia are the top countries producing dates and showed a growth trend of 222 and 197%, respectively, from 1990 to 2021 [20]. On the other hand, due to such a huge growth production trend, large quantities of freshly harvested dates can be lost during the harvest, storage, or processing stage, representing major concerns for date fruit cultivators. Lost stocks are mostly used for animal feed due to their undesirable and damaged texture [21]. Therefore, the application of dates in food products such as snack bars can boost the products’ functional properties and serve as an alternative to dates’ direct consumption, reducing the lost fresh stock. In this review, studies showing the potential application of date fruit for developing different types of enriched snack bars is discussed regarding their nutritional, chemical, functional, and sensory attributes. Research articles were screened from PubMed, ScienceDirect, and EBSCO, with a further search in Google Scholar, using the keywords “date fruit”, “*Phoenix dactylifera*”, “date bars”, “energy bars”, “snack bars”, “date energy bars”, and “date snack bars”.

## 2. Date Fruit

Date fruit (*P. dactylifera*) is one of the most important crops of the Arecaceae family; it has been grown since ancient times, more than 4000 years ago, mainly in Middle Eastern and Northern African countries, predominantly in dry and arid regions. It has been cultivated primarily for cultural, nutritional, environmental, religious, and social development purposes [22,23]. Dates can be found in numerous varieties, with more than 600 grown worldwide, such as Ajwa, Barhe, Halawi, Khlas, Lulu, Medjool, and Sukkari, which differ from each other in shape and organoleptic properties. Dates can be classified based on their ripening stages into five primary types: Hababouk, Kimri, Khalal or Bisr, Rutab, and Tamer. All five stages may be processed and consumed in different ways; several kinds of food products can be processed from the different date stages, such as dried dates, date paste, date syrup, date jam, date butter, and pickled dates. Moreover, date wastes can be processed into different by-products, such as the processing of date pits—seeds—into coffee and oil [5,22,24,25].

### 2.1. Production of Date Fruit

**Figure 1:**
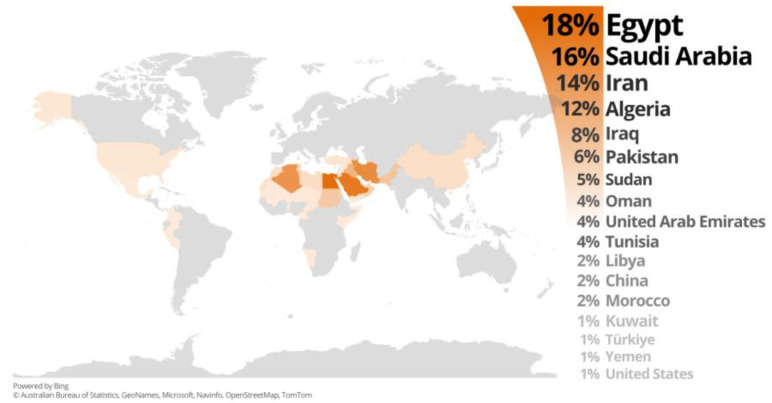
Top countries producing date fruit and their share (%) of the total world’s date production according to the Food and Agriculture Organization statistical data in 2021 [20].

Dates are considered highly valuable from environmental and economic aspects [5,22]. The world’s date production has markedly increased from 3.4 million tonnes produced in 1990 to 9.6 in 2021, representing 181% growth. As shown in Figure 1, the leading producer country is Egypt, followed by Saudi Arabia, with more than 1.7 and 1.5 million tonnes produced in 2021, respectively, accounting for 18 and 16% of the total world’s production and representing significant increases of 222 and 197% from 1990 to 2021, respectively. The higher production in Egypt is mainly attributed to its significantly higher tree density per hectare compared with that of Saudi Arabia. The next leading producers ranking among the top 10 countries producing dates after the first two are Iran, Algeria, Iraq, Pakistan, Sudan, Oman, United Arab Emirates, and Tunisia, sharing 55% of the total world’s production. Other countries share 5% of the world’s date production, with Libya, Morocco, Kuwait, Türkiye, and Yemen each producing 1–2%, ranging from nearly 60,000 to more than 179,000 tonnes produced in 2021 [20].

The cultivation of dates has also moved across the world with the development of civilization in other regions, such as European, American, and East Asian countries; China shares 2% of the total world’s production, with more than 159,000 tonnes produced in 2021, while 1% is from the United States, with more than 53,000 tonnes produced in 2021. Other European and American countries such as Albania, Mexico, and Peru share less than 0.5% of the world’s production, with roughly 14,000, 19,000, and 300 tonnes produced in 2021 [20].

### 2.2. Nutritional Characteristics of Date Fruit

The nutritional composition of date fruit mainly consists of carbohydrates, fiber, and protein with low amounts of fat (Table 1). Excellent amounts of micronutrients are also present, including vitamins such as thiamine, riboflavin, C, and E; minerals such as potassium and magnesium; and different phytochemicals. This section provides an overview of the nutritional characteristics of different date varieties grown worldwide.

#### 2.2.1. Carbohydrates

Based on dry weight, date fruit mainly comprises carbohydrates, ranging from 40 to more than 80%, depending on the cultivated variety. Several studies have evaluated various date varieties, and among the large range of more than 600 varieties grown worldwide, the highest total carbohydrates, more than 80%, were found in Burni. At the same time, the lowest was less than 50% in Khasab [5]. Other date varieties containing high total carbohydrate content after Burin, ranging from 71 to 79%, are Ajwa, Khodari, Labanah, and Sukkari [6]. Dates’ carbohydrates are mainly reducing sugars and non-reducing sugars with small amounts of polysaccharides; the reducing sugars are present in the form of glucose, fructose, mannose, and maltose, while the non-reducing sugars are primarily sucrose, and the polysaccharides are mainly cellulose, starch, and β-glucans [8]. Dates’ glucose, fructose, and sucrose were found to highly vary in their amounts, ranging from 2 to 95 mg 100 g^−1^, depending on the date variety; the highest glucose content of 95.4 mg 100 g^−1^ was found in the Khalas variety cultivated in Al-Kharj farms in Saudi Arabia. The same variety cultivated in other Saudi farms, such as Al-Qassim and Al-Ahsa, was recorded to have a glucose content of 79.6 and 58.2 mg 100 g^−1^, respectively. Higher fructose contents of 113, 101, and 74 mg 100 g^−1^ were also found in this variety cultivated in Al-Kharj, Al-Qassim, and Al-Ahsa, respectively, with lower amounts of sucrose ranging from 17 to 31 mg 100 g^−1^ [8]. Other date varieties, such as Ajwa, were consistently found to vary in glucose, fructose, and sucrose contents in amounts ranging from 35 to 54.5, 39 to 52.5, and 0 to 13.4% [5].

These examples of dates’ carbohydrate contents indicate significant variation among dates depending on the variety and geographical conditions. Other factors, such as the ripening stage, can also significantly affect carbohydrate content; as dates begin to ripen, increases in the activities of multiple hydrolyzing enzymes, such as pectinase, are observed, leading to the degradation of dates’ pectin content, generally ranging from 0.5 to 4% [27,28]. On the other hand, the different analysis methods applied by various researchers can also affect the obtained results, leading to an invalid quantitative comparison between date varieties. However, date fruits are generally considered rich in carbohydrates [5].

#### 2.2.2. Dietary Fiber

Date fruit is considered a rich source of dietary fiber, ranging from 2 to 8%; the highest fiber content of 8% was found in the Deglet-Noor variety, mostly cultivated in Algerian and Tunisia. The well-known Moroccan date variety “Medjool” also contains a high amount of fiber, reaching 6.7%. In comparison, the lowest amount of 2.7% was found in the Lulu variety from the United Arab Emirates [7]. Median amounts of fiber of 4.35% were found in Sukkari dates cultivated in Saudi Arabia [26]. Dates’ dietary fiber consists of good-quality fiber fractions, such as β-glucans, arabinoxylans, and cellulose, providing an excellent source of dietary fiber, even better than that of cereals [5].

#### 2.2.3. Lipids

Dates contain extremely low amounts of fat, less than 1% of the total fruit; a maximum fat content ranging from 0.5 to 0.7% was found in three cultivars from Saudi Arabia, Sukkari, Burni, and Labanah, while a minimum of 0.1 to 0.2% was found in other Saudi varieties, Khodari and Mabroom [6,23]. Although dates’ nutritional composition may vary depending on the fruiting stage, fresh dates contain 0.1–0.2% fat, while dried dates contain 0.1–0.5% [25]. Significantly higher amounts of fat, from 7 to 9.7%, were found in date seeds or pits [1]. Others reported fat content reaching 13% [29]. The date pit’s fat content consists of high-quality saturated and unsaturated fatty acids, such as lauric, palmitoleic, oleic, linoleic, and linolenic acids. The oleic acid content in the pit can be found in amounts ranging from 41 to 58.8% [29,30], while lauric and linoleic acids in some date varieties were found in 17.8 and 15% of the total pit’s oil content. The unsaturation degree of date pits’ oils is less than that of common olive oil. However, oils with high oleic acid contents are generally considered high quality due to their high stability and nutritive importance [29]. High oleic oil content has been recognized as highly nutritious due to its beneficial effects against cardiovascular disease by lowering total cholesterol and low-density lipoprotein cholesterol [31]; therefore, date pits are considered a potential source for such fatty acids and have been used in multiple studies for developing potential functional food products or as a replacement for commercial oils [32,33].

#### 2.2.4. Proteins

The protein content in dates is considered low, ranging from 1.7 to 4.7%, although such concentrations are higher than that in other fruits [1,6,25]. A maximum protein content of 4.7% was found in the Saudi variety Shalaby, while a minimum of 1.7% was found in another Saudi variety, Mabroom [6]. Median amounts of protein ranging from 1.6 to 3.64 of crude protein were found in date varieties such as Khalas, Barhi, Lulu, Deglet-Noor, Medjool, and Qush Balquan [5]. The protein content may also vary depending on the dates’ fruiting stage; amounts ranging from 1.1 to 2.0% were found in fresh dates, while dried dates showed amounts ranging from 1.5 to 3.0% [25]. Such protein profiles were reported to have molecular weights ranging from 12,000 to 72,000 Dalton, though most date varieties contain two prominent bands appearing at 30,000 and 72,000 Dalton [34].

Dates’ amino acid profile contains high-quality acids, including nonessential and essential acids such as proline, lysine, histidine, tyrosine, isoleucine, and tryptophan. Lysine, a key essential amino acid lacking in most cereals, has been found in concentrations ranging from 0.025 to 0.073% in different date varieties, with the maximum content found in Ajwa dates. Some of the presented acids may also be lacking in common fruits such as oranges, bananas, or apples; isoleucine, for instance, is 800 times higher in dates than in apples, while lysine is nearly 2000 to 5000 times higher than in apples, bananas, and oranges [7].

#### 2.2.5. Vitamins and Minerals

Dates are considered a relatively good source of different vitamins (Table 2), mainly B complex; thiamine, riboflavin, and niacin were found in some date varieties at 0.050–0.66, 0.060–0.66, and 1.27–1.61 mg 100 g^−1^, respectively [7]. Other studies reported relatively high contents of tocopherols, a form of vitamin E, found in 12 different Saudi date varieties in amounts ranging from 0.07 to 0.21, 0.01 to 0.03, and 0.01 to 0.04 ng 100 g^−1^ based on fresh weight for α-, β-, and γ-tocopherol, respectively [8]. Date pit oil was also found to contain high amounts of tocopherols, including α-, β-, γ-, and δ-tocopherols, ranging from 44.73 to 110.82 mg 100 g^−1^ depending on the date variety [35]. Some date varieties may also contain vitamin C in amounts that are relatively small but higher than those found in dried fruits such as apricots, figs, and raisins. The relative amounts of vitamin C in these fruits range from 0.002 to 0.02% (2.4–17.5 mg 100 g^−1^) [1,25,28]. Vitamin A was found in dates in amounts averaging 0.001 mg 100 g^−1^ [28]; the major forms of vitamin A, carotenoids, present in dates are lutein and β-carotene [5]. Vitamin K was also found in some date varieties at 2.7 μg 100 g^−1^ [34]. However, dates’ vitamin content can significantly vary between the fresh and dry stages due to vitamin depletion during drying [1,25].

Minerals such as potassium, calcium, and magnesium are found in relatively high amounts in dates, ranging from 0.05 to 0.9%. The most abundant element found in dates is mostly potassium, followed by calcium. Some date varieties, such as Sukkari, contained higher mineral amounts than other fruits, such as pomegranate or mango [23]; others reported that some dates contain 2.5 times higher potassium than bananas [25]. Elements such as iron, phosphorus, sodium, and copper are found in lower amounts, ranging from 0.0049–0.027%, with sodium being the least abundant [6,23]. The high potassium and low sodium contents in dates make them excellent products for hypertensive individuals [36].

#### 2.2.6. Phytochemicals

Many phytochemicals, such as carotenoids and phenolic compounds, are present in dates in relatively high amounts. Phytochemicals are known for their strong antioxidant capacities; their presence in any food product can greatly boost its functional properties. Total phenolic compounds, including flavonoids and nonflavonoids, can be found in dates in amounts ranging from 26.47 to more than 27,000 mg gallic acid equivalents (GAE) per 100 g^−1^ (Table 2). High amounts of 26,000–27,000 mg GAE 100 g^−1^ were found in Lulu and Khenaizi varieties, commonly consumed in Malaysia [12]. Some flavonoid compounds, such as anthocyanins, are involved in the date pigmentation process by providing a dark-red pigment, and they undergo degradation after the later ripening stages [27].

The total phenolic content can significantly vary between fresh and dried dates; the Khalas variety, for instance, contained 134 mg ferulic acid equivalents (FAE) per 100 g^−1^ while fresh, but after drying, the content increased to 339 mg FAE 100 g^−1^ [37]. Phenolic compounds can also vary significantly depending on the date variety, the growth or maturation stage, and geographical conditions. For instance, high amounts of polyphenols were detected in different date varieties but declined after date ripening [5].

Several detected phenolic compounds were found in various date varieties; different phenolic acids were detected in Ajwa dates, including ferulic, hydroxybenzoic, gallic, caffeic, vanillic, chlorogenic, isovanillic, chlorogenic, protocatechuic, isoferulic, and hydroxycinnamic acids [38]. Other date varieties, such as Sukkari, Mabroom, Khalas, and Nabtat-Saif, were reported to contain different flavonoid compounds, such as quercetin, luteolin, apigenin, isoquercetrin, and rutin, in amounts ranging from 1.2 to 2.8 mg 100 g^−1^ for total flavonoid compounds [8]. Such a high and wide variety of phenolic compounds can play a major role in elevating the antioxidant potential by inhibiting lipid peroxidation; the evaluation of the antioxidant activities of multiple date varieties revealed high antioxidant capacities ranging from 55 to 75% DPPH (1,1-diphenyl-2-picrylhydrazyl) scavenging/inhibiting activity [12]. Others reported that the DPPH inhibition activity of nine different date varieties ranged from 22 to 40% [39]. Dates also contain other major classes of phytochemicals with high antioxidant capacities, such as carotenoids and phytosterols; several other studies have reported the strong antioxidant capacity of dates cultivated in different countries, such as Algeria, Kuwait, Oman, Bahrain, and the United States [5].

**Table 2:** Vitamins, minerals, and total phenolic content in different date fruit varieties.

	Range	Reference
Vitamins		
B complex (mg 100 g^−1^)		
Thiamine	0.050–0.66	[7]
Riboflavin	0.060–0.66	
Niacin	1.27–1.61	
Vitamin E (ng 100 g^−1^)		
α-Tocopherol	0.07–0.21	[8]
β-Tocopherol	0.01–0.03	
γ-Tocopherol	0.01–0.04	
Vitamin C (mg 100 g^−1^)	2.4–17.5	[1,25,28]
Vitamin A (mg 100 g^−1^)	0.001 *	[28]
Vitamin K (μg 100 g^−1^)	2.7 *	[34]
Minerals (mg 100 g^−1^)		
Potassium	289.6–2220	[7]
Calcium	39–187	
Magnesium	43–150	
Sodium	1–8.9	
Phosphorus	12–64	
Iron	0.9–4.94	
Zinc	0.29–0.65	
Total Phenolic Content		
(mg GAE 100 g^−1^)	26.47–27,106.76	[12]
(mg FAE 100 g^−1^)	134–343	[37]

Abbreviations: * Average; GAE, gallic acid equivalents; FAE, ferulic acid equivalents.

### 2.3. Functional/Pharmacological Effects of Date Fruit

#### 2.3.1. Antioxidant Effects

Elevating the body’s antioxidant system, or in other words, eliminating oxidative stress, a state caused by the excess production of free radicals leading to the development and progression of various diseases, can be achieved by consuming dates. The rich contents of different date phytochemicals were significantly correlated in some studies with dates’ antioxidant potential [11,24]. For instance, phenolic acids present in dates can act as strong free radical scavengers; in vivo studies have shown that the intake of some dates’ phenolic acids, such as gallic and ferulic acids, increased antioxidant enzymes in animal models’ cardiac tissue [10]. Other in vivo studies performed in various toxicant-induced animals showed that date extracts at concentrations ranging from 100 to 1000 mg kg^−1^ of body weight (BW) were significantly effective in improving the levels of antioxidant markers, including glutathione transferase, catalase, glutathione reductase, and malonaldehyde [40,41,42,43]. Date pits can also promote a strong antioxidant capacity; in a recent double-blind, randomized, controlled trial, the consumption of 26 g of date seed powder before workouts daily for two weeks showed positive effects by improving different oxidative stress markers, such as superoxide dismutase, glutathione peroxidase, and malondialdehyde [44].

#### 2.3.2. Anti-Inflammatory Effects

Besides elevating the antioxidant system, date intake can also reduce the body’s inflammatory state, another critical system in the development and progression of various diseases. A reduction in the inflammatory state is also attributed to dates’ strong antioxidant potential; inflammatory states are mostly initiated due to oxidative stress due to free radicals [11]. The administration of 5 g kg^−1^ BW of date pit powder to carbon tetrachloride (CCI_4_)-induced rats for two weeks resulted in significant improvements in the levels of some pro-inflammatory cytokines, such as tumor necrosis factor (TNF-α) and interferon-gamma (IFN-γ) [45]. A systematic review that evaluated pro-inflammatory cytokines such as TNF-α and interleukins (IL) showed improvements after consuming date pits [14]. Other studies reported consistent results after consuming either dates or their pits [13,46,47,48,49].

#### 2.3.3. Anti-Hyperglycemic Effects

Despite the rich contents of sugars in dates, some in vivo studies have reported hypoglycemic effects after the intake of dates; El Abed et al. [50] showed that the administration of an aqueous ethanolic data extract at 200 mg kg^−1^ BW to animal models was effective in alleviating postprandial glycemia. These positive effects were attributed to inhibitory activities against some enzymes related to type 2 diabetes, such as α-glucosidase, an intestinal enzyme responsible for regulating glucose availability for intestinal absorption [51]. In a recent in vivo study, alloxan-induced diabetic rats were administered Ajwa date pulp or pit powders at 7 and 1.5 g per 100 g of rat chow for four weeks. The results showed that Ajwa date pits significantly alleviated blood glucose levels compared to controls. In contrast, date pulp did not show such positive results, indicating the significant antihyperglycemic effect of date pits [52]. Another recent in vivo study performed in dexamethasone-induced diabetic rabbits also showed that the oral administration of a date pit extract at doses ranging from 200 to 400 mg kg^−1^ BW resulted in antidiabetic effects based on improvements in glucose uptake stimulation, cellular glycogen synthesis, and/or pancreatic cell protection against dexamethasone injection [53]. Date intake in the form of whole fruit may show significant antidiabetic effects depending on the date variety; in a systematic review and meta-analysis, it was reported that the intake of different date varieties among diabetic individuals was effective in alleviating glycemia [54]. However, this systematic review only included five research studies; thus, a limited number of studies cannot confirm the significance of such results, as agreed by Mirghani [54]. In addition, a randomized control trial performed on type 2 diabetic patients showed that the daily intake of 50 g of Lulu dates synergically with standard oral antidiabetic drugs for two weeks had no alleviating effect on blood glucose levels compared to the control group [55]. Therefore, diabetic patients may need to consume dates consciously, and further studies regarding dates’ hypoglycemic effects are needed to confirm such claims.

Though date fruit is generally considered low in glycemic load, a study examining the glycemic index (GI) of five different date varieties in healthy individuals showed that the intake of 50 g of dates with available carbohydrates equivalent to 50 g of glucose had an average GI ranging from 30 to 69 [56]. In another prospective clinical trial on healthy individuals, based on the intake of 50 g of 17 different date varieties in the Tamar stage, GIs ranged from 42.8–74.6, with two varieties, Ajwa and Shagra, having the lowest values of GIs, 8.5 and 9.2, respectively [57]. Regardless of the dates’ ripening stage, other trials showed that both Bisr and Tamar stages had similar GIs of 54–55 on average [58]. On the other hand, dates’ high fiber and polyphenol contents could impact glycemia by improving the gut microbiome profile linked to the onset of type 2 diabetes [59]. Polyphenols are shown to interact in a variety of enzymatic processes mediated by the gut microbiome [13]. At the same time, fiber is broken down by bacterial fermentation, generating short-chain fatty acids as by-products that promote the expression of several transporters responsible for glucose homeostasis [60].

#### 2.3.4. Anti-Hypercholesterolemia Effects

Due to their rich dietary fiber content and various bioactive phytochemicals, dates were also reported to promote cholesterol-lowering activities. Date extracts at concentrations ranging from 125 to 1000 mg kg^−1^ BW were orally administered to hypercholesterolemic rabbits for 10 weeks. Supplementation with date extracts led to enhancements in lipid metabolism based on reduced levels of total cholesterol, low-density lipoprotein (LDL), and triglycerides. In contrast, the high-density lipoprotein (HDL) level was increased compared to the control group [61]. In another study, the administration of 300 and 600 mg kg^−1^ BW of a date suspension for 14 days led to reductions in the levels of total cholesterol, triglycerides, and LDL, while levels of HDL showed an increase, indicating the anti-hypercholesterolemic effects of dates [62]. Date pits were also reported to possess effective cholesterol-lowering properties; in diet-induced hypercholesterolemic rats administrated date pit extracts at concentrations ranging from 0.25 to 1 g kg^−1^ BW for 21 days, the results showed significant dose-dependent enhancements in lipid metabolism based on reduced levels of total cholesterol and LDL as well as the atherogenic index [63]. The cholesterol-lowering properties of dates may significantly vary depending on the date variety; it was shown that Hallawi and Ajwa, compared to Aseel and Khudravi varieties, were more effective in enhancing lipid metabolism in animal models [19].

#### 2.3.5. Anti-Tumor Effects

Discovering effective anti-tumor agents is of major interest in the research field due to the numerous adverse effects of existing cancer treatments, such as radiotherapy and chemotherapy. The functional properties derived from fruits’ phytochemicals, such as flavonoids and other phenolic compounds, have been linked to anti-tumor activities [64,65]. Date fruit has been demonstrated to have strong anti-tumor potential due to its rich contents of various bioactive phytochemicals; for instance, the bioactive compounds rutin and quercetin derived from Ajwa dates were orally administrated to breast tumor mutagenic mice at a dosage of 5 mg kg^−1^ BW for 11 days synergically with an injection of doxorubicin [66], a frequently used chemotherapeutic agent against breast cancer known to cause cardiotoxic effects. This intervention led to positive effects based on the alleviation of weight loss caused by the doxorubicin injection and a significant reduction in the levels of plasma cardiac troponin-I, a protein biomarker used to diagnose heart attacks [67]. The intake of Ajwa dates during the standard treatment of pediatric cancer patients was shown to promote significant improvements in patients’ treatment outcomes; they had lower hospital admissions for fever-associated neutropenia and lower infection risks than the control group. In contrast, patients who did not have Ajwa dates showed a higher mortality rate, mainly due to disease progression associated with infections [68]. In another recent intervention using rat models induced with hepatocellular carcinoma [69], the daily oral administration of Barhi date extracts at 400 mg kg^−1^ BW promoted anti-tumor activities mediated by cell proliferation inhibition. It was shown that the Barhi date extract exerts inhibitory effects on critical signaling pathways related to cellular growth and functions, such as PTEN and AKT, namely, *phosphatase* and *tensin homolog* and *protein kinase B*, respectively. Date leaf extracts were also reported to promote significantly stronger anti-tumor activities than date pulp or pit extracts; the results showed that the phenolic contents and antioxidant capacity of the date leaf extract were significantly higher than those of the pulp or pit [70]. Consistent positive results were observed in several in vitro studies with extracts of different date varieties or different date parts, including pits and leaves [15,16,71,72].

#### 2.3.6. Other Effects

Various pharmacological effects of date fruit were also reported in several studies. The high potassium content in dates can significantly benefit hypertensive individuals; the role of potassium in regulating blood pressure is well established [73]. Dates also contain other bioactive compounds that manage hypertension, such as lauric acid, linolenic acid, palmitic acid, tocopherols, β-sitosterol, and isosorbide [17]. Obode et al. [17] reported the in vitro antihypertensive effects of date extract mediated by inhibitory activity against angiotensin-converting enzyme, a key enzyme in controlling blood pressure by regulating the body fluid volume. The antihypertensive properties of dates can also promote significant positive effects on pregnant women, as their blood volume expands during pregnancy [22]. Date intake during pregnancy was also reported to reduce the gestation duration and accelerate natural delivery [74]; in a systematic review and meta-analysis of clinical trials examining the effects of date consumption on pregnant women, it was concluded that women who consumed 50–100 g of any form of date fruit daily from 36–38 weeks until delivery had a significantly shorter duration of the first stage of labor [68].

Moreover, dates were reported to promote antimicrobial effects; different date varieties’ extracts were found in vitro and in vivo to exert inhibitory effects against Gram-positive and Gram-negative bacteria, such as *Bacillus subtilis*, *Salmonella typhi*, *Enterococcus faecalis*, *Escherichia coli*, *Bacillus cereus*, and *Staphylococcus aureus* [5]. Inhibitory effects against the growth of fungal strains such as *Fusarium oxysporum* were also reported after using date extract, indicating the potential usage of dates against food spoilage microbes [18]. Furthermore, date pit extract was shown to promote anti-viral effects against a *Pseudomonas* phage at a relatively extremely low concentration of less than 10 mg ml^−1^; such inhibitory effects were likely attributable to the date pit extract’s ability to bind directly to phages [75]. In another study, an ethanolic date extract was used to formulate a cream-based treatment against herpes simplex virus type 1 (HSV-1), a member of the *Herpesviridae* family known to cause a range of partly life-threatening infections in humans and animals and considered a significant public health concern. The ethanolic date extract at a 5% concentration showed more effective and faster anti-viral activities than other concentrations compared to acyclovir, a reference drug used for treating HSV-1 infections [76]. However, studies regarding this issue are limited, and further investigations are needed to provide more evidence on dates’ anti-viral potential.

## 3. Date Bars

The application of different types of fruits for developing snack bars is widely used; fruit-based bars have been developed to provide excellent snack bars containing natural sugars, vitamins, and minerals with other bio-nutritive components to meet consumers’ required daily nutritional intake [1]. Date fruit can be a favorable choice in developing snack bars due to its highly valuable nutritional and functional attributes; dates were first explored for developing energy bars back in the 1970s when Kamel and Kramer [77] performed a pilot study aiming to use dates, as a high-carbohydrate and low-protein fruit, to develop snack bars due to their widespread cultivation in some areas where protein shortage was persistent. Since then, protein enrichment has been the main target in preparing and producing date-based bars.

### 3.1. Possible Functional Ingredients Used in Date Bars

Depending on the manufacturing target, date bars can be formulated with only one or two ingredients or with many several ingredients. Regarding protein enrichment, many different protein sources can be used to prepare date bars, including either dairy or plant sources. Proteins include milk powders or its products (e.g., whey concentrates and isolates), soy proteins, or other plant-derived proteins (e.g., sunflower and vetch). Many other ingredients can also be incorporated to deliver nutritionally balanced, functional, flavorful, and enjoyable date bars, including cereals (e.g., oats, wheat, rice, or barley), legumes (e.g., chickpea and soybean), dried fruits (e.g., cherries, apricots, apples, or pears) and nuts (e.g., almonds, peanuts, walnuts, or pistachios) with the addition of small amounts of spices for seasoning (e.g., salt, cardamom, cinnamon, or ginger). These different ingredients can deliver functional attributes; other novel ingredients can also deliver much stronger functional attributes, such as plant seeds and fruit polyphenol extracts [78,79,80,81,82,83,84].

### 3.2. Formulation of Date Bars

Several researchers worldwide have prepared multiple variations of date-based bars (Table 3); this section summarizes their findings and their suggestions for preparing excellent formulations of date bars rich in nutrients and other bioactive components to be consumed by athletes and children or as sweet/dessert substitutes for regular consumption. In a pilot study, Kamel and Kramer [77] prepared a simple and basic date bar recipe using skim milk powder and single-cell protein with walnuts. Significant increases in protein content of 130–175% were recorded depending on the added concentrations of protein sources. Protein availability also showed an increase of two–three-fold. With protein contents of 56 and 37%, respectively, single-cell protein and dry skim milk are rich sources of protein. Single-cell protein is derived from food-grade cultivated microbes and algae, mainly those containing more than 30% proteins in their biomass [85]. Walnuts were mainly incorporated for taste, although nuts are known for their rich oil contents, mainly unsaturated fatty acids [86]. The addition of walnuts led to a significant increase in fat content of 300% resulting in an increase in the energy value from 364 to 407–413 Cal 100 g^−1^. This formulation of date bars also had excellent amounts of minerals and vitamins. Increases in calcium, iron, phosphorus, and magnesium of 30, 26, 150–250, and 13–35%, respectively, were observed compared to control bars. High phosphorus and magnesium contents were mostly derived from single-cell protein, while calcium was from dry skim milk. Single-cell protein also contains good amounts of vitamins such as thiamin and riboflavin, which were significantly increased in the bars. However, after 6 months of storage at ambient temperature (25–37 °C), there was a loss in both vitamins of 10–25%, while storage at 5 °C maintained both vitamins’ contents, indicating the importance of the storage time and temperature in maintaining their contents. These two factors can also affect the bars’ sensory quality; bars stored at 5 °C had higher scores for flavor and texture compared to those stored at higher temperatures. However, storage at ambient temperature for 3–6 months led to significant improvements in the overall sensory quality of bars made from dates alone (control bars) compared to their initial quality, while enriched bars stored under the same conditions showed the opposite effect.

Yeast-derived proteins such as Toruway and Zyest were used in another study in the 1980s. In a similar approach to that used by Kamel and Kramer [77], Sawaya and Khalil, with their colleagues [87,88], used soy protein isolate and skim milk powder with the addition of yeast proteins. Half of the bars were dipped in milk chocolate, producing two bars, plain and chocolate. This formulation significantly increased the protein content by 72–128%, with soy protein isolate contributing the highest content. Compared to the control, a more balanced amino acid profile was recorded in the fortified bars. Soy protein isolate and skim milk powder added at 1.5 and 10.5%, respectively, were indicated as the optimal levels for sufficient protein content. Dipping the bars in milk chocolate also led to a relative improvement in protein availability based on the presence of lysine, which was not found in the control plain bars. However, the protein quantity was not improved by dipping the bars in chocolate, indicating protein fortification’s significance.

Moreover, soy protein isolates and skim milk powder contain higher amounts of minerals than dates. Major increases (2–4 times) in several minerals, such as sodium, potassium, and calcium, were observed in fortified bars. The addition of these ingredients caused nonsignificant changes in overall sensory acceptability compared to control bars. Similar to Kamel and Kramer’s results [77], storage time and temperature were indicated as key factors affecting sensory quality. After three months of storage at 7 °C, fortified bars showed significantly lower sensory scores in terms of taste compared to controls. Since then, the protein fortification of date bars using single-cell protein has not been applied much. In several later studies, milk powder was commonly used due to its promising effects in the food industry [89]; the addition of skim milk powder with other ingredients, including 7–11% oat flakes, 6% sesame seeds, and almonds, has been explored [90]. Increases in protein content of 17–36% were recorded, with the highest content in bars containing 12% skim milk powder. Protein availability also showed improvements based on an increase in essential amino acids such as lysine, histidine, and phenylalanine of 49.5, 41, and 26%, respectively. Skim milk powder also led to decreases in fat content of 35–66%. Enhancements in mineral profiles were also recorded; higher amounts of minerals such as phosphorous, calcium, and potassium were recorded with higher concentrations of skim milk powder.

However, these enhancements can depend on the used date varieties. Al-Hooti and his colleagues [90] used four different date varieties, Bushibal, Gash-Gaafar, Lulu, and Shahla, from the United Arab Emirates. They observed some differences in mineral contents depending on the date variety. In another study using different date varieties, Nabtat-Ali and Sukkari showed significantly different effects on proximate composition [91]. Although significantly higher protein content was achieved due to milk powder fortification, Sukkari bars were recorded to have 9% higher protein content than Nabtat-Ali bars. Nabtat-Ali bars showed 18% higher fiber content than Sukkari bars. Sukkari dates also had a 6% increase in total phenolic content (240.33 vs. 224.33 mg GAE 100 g^−1^ for Sukkari bars vs. Nabtat-Ali bars, respectively). However, raw dates showed higher antioxidant activity than date bars; exposure to higher temperatures during the preparation processes applied in this study, such as heating or boiling, could significantly reduce the total phenolics. Bars’ sensory quality can also differ based on the variety used; Sukkari bars were tenderer or softer than Nabtat-Ali bars, and the latter required twice the time for chewing. Sukkari dates also contributed to a better color. However, neither bar received higher scores in flavor or overall acceptability, indicating that additional work, such as adding suitable flavoring agents, is needed.

Other formulations of date bars using skim milk powder at different concentrations and using other ingredients were also explored. The addition of 20% skim milk powder, soybean flour, or almond flour was explored [79]. Between the different formulations, bars made with soybean flour exhibited higher protein content with good amounts of fiber. Soybean flour contributed to a highly significant increase in protein content of more than 300%, followed by almond flour and skim milk powder, with increases of 182 and 142%, respectively. Almond flour also contributed to significantly higher contents of fats and fiber, while soybean flour and skim milk powder showed slight decreases in fats of 13–20%. Fiber content was the lowest in bars made with skim milk powder, even lower than in control bars. Thus, skim milk powder is a good choice for sufficient protein content, but higher protein content can be achieved using soybeans. It also requires fortification with other nutrient sources to maintain sufficient fiber and fats.

On the other hand, the date fruit used in [79] had a good content of vitamin C (10.67 mg 100 g^−1^); in the early stages of date maturity, vitamin C can be found in high amounts, but it shows a decline during maturation [92]. The addition of soybean flour increased vitamin C by 8%, whereas skim milk powder and almond flour decreased by 28% and 13%, respectively. However, bars made with soybean flour were the most disliked in taste, while those containing almonds were the most liked. Nuts have a high-intensity taste due to their dense nutrients. Thus, adding nuts can strongly enhance the bars’ sensory quality [86]. The addition of soy with other flours combined with a low concentration of skim milk powder was used in another study [83]; chickpea, oat, and soy flours were added at consistent concentrations with 3.5% of skim milk powder. This formulation showed high contents of protein, fats, and fiber; oat flour contributed the highest amounts of fiber due to its richness in good-quality fiber, such as β-glucans. At the same time, soy and chickpea flours contributed higher contents of proteins. These ingredients also showed significant increases in total phenolic content; bars containing a combination of all ingredients had the highest total phenolics. The combination of these ingredients also resulted in the highest overall sensory acceptability. In a similar formulation of date bars, Munir et al. [82] used roasted oatmeal flour, roasted chickpea flour, skim milk powder, almonds, pistachios, and cardamom for seasoning with the addition of Carboxy Methyl Cellulose, a thickening and binding food additive. All ingredients were constant, with varied concentrations of oatmeal flour. Significant increases in protein, fat, and fiber contents of 24–47, 116–176, and 62–94%, respectively, were recorded depending on oatmeal concentrations. The mineral profile also showed improvements due to oatmeal addition; increases in magnesium, zinc, and manganese of 62–162, 52–75, and 141–193%, respectively, were recorded.

Adding cereals such as oatmeal can also significantly decrease the moisture content, leading to higher shelf-life stability by limiting microbial growth. Such ingredients can decrease the moisture content by binding water to their fractions, leading to lower water activity (^a^_W_). An ^a^_W_ below 0.6 is critical in manufacturing food products to ensure limited microbial growth. Munir et al. [82] recorded A_w_ values ranging from 0.56 to 0.59 depending on the oatmeal concentration. The lowest ^a^_W_ was recorded with a higher amount of oatmeal. Lower moisture content can also affect the bars’ texture; an increase in the bars’ hardness to an acceptable range was observed. In contrast, the use of chickpea flour in other studies led to higher moisture content, resulting in extremely soft date bars compared to date bars containing corn flour. The latter was more liked in terms of texture and overall sensory acceptability [84]. Therefore, choosing between cereals, legumes, flours, and any other dry ingredients is critical to maintaining a lower moisture content when considering the addition of protein ingredients.

The addition of varied concentrations of extruded rice wheat, extruded wheat, extruded barley, or popcorn with date syrup and sesame seeds was explored [21]. Bars containing 30% portions of cereal showed significantly higher hardness scores due to a decrease in moisture content. Cereals can absorb date syrup, leading to decreased moisture content; hence, bars become more compressed. Regarding taste, appearance, color, and overall sensory acceptability, bars containing extruded wheat scored the highest, while the mixture of extruded cereals scored the lowest. Cereal addition also increased protein by 31%, mainly derived from extruded wheat and barley. Fiber content was also higher due to its richness in extruded barley.

On the other hand, date syrup and sesame seeds are rich in fat content, leading to higher sensitivity to oxidation. Fortification with essential oils such as thymol for improving oxidative stability was explored [93]; thymol was added with encapsulation to overcome its strong unpleasant taste and odor. This approach improved the bars’ oxidative stability more efficiently than butylated hydroxytoluene, a known phenol derivative used for antioxidant properties in the food industry.

Other ingredients, such as dried fruits, can also affect date bars’ moisture content; dried apricot paste was explored with other ingredients, including chickpea flour, roasted peanuts, and skim milk powder [94]. Depending on the apricot paste concentration, the moisture content was increased to acceptable ranges of Aw between 0.53 and 0.55 with increased apricot contents. Bars’ sensory quality was also affected by the addition of apricots; darker colors were observed with higher concentrations of apricots. The softness of the bars was also increased due to their increased moisture content. The bars’ bioactivity was also enhanced by higher apricot concentrations based on significant increases in total phenolics, ranging from 225.20 to 263.84 mg 100 g^−1^. Higher free fatty acid contents ranged from 0.060 to 0.081 g 100 g^−1^.

Protein fortification can also be applied using different protein sources other than milk powder or its products; cheddar cheese and whey protein isolate were recently explored with other ingredients, including roasted chickpea flour, roasted rice flour, and dried apricots [4,78]. Dates and apricots mostly contributed to higher levels of bioactive components, while whey protein and cheddar cheese delivered higher protein content. Adding cereals or legume flour also increased protein by 3–6%, with chickpea flour having a higher contribution than rice flour. Bars’ bioactive components in total phenolics increased dramatically due to their richness in dates and apricots. Cheddar cheese also contributed relatively high levels of total phenolics, possibly due to cheese’s macro-components’ ability to release some bound phenolics. However, higher concentrations of cheddar cheese led to the lowest contents of total flavonoids; in contrast, bars containing more whey protein, apricots, and dates contained higher levels of flavonoids. Dates generally contain thirteen flavonoids, such as quercetin and catechin, that can strongly interact with whey protein components, leading to a significant decrease in the total content. Accordingly, higher antioxidant activity was recorded in the latter bars; dates and apricots are also rich in vitamins and minerals that either directly scavenge free radicals or contribute to stimulating the body’s endogenous antioxidant system.

Nonetheless, fortification with cheddar cheese can deliver significantly higher protein content than other bioactive ingredients. However, the cheddar bars in this study were highly disliked in terms of flavor and overall sensory acceptability, although the unaccepted cheese flavor depends on regional preferences. Thus, as stated by the authors, Pakistani folks are not used to cheese flavor. Choosing an excellent protein source that does not negatively affect the bars’ sensory quality can be challenging. Sunflower protein concentrate and isolate were used in a recent study and scored the lowest overall sensory quality; control bars were more liked than those fortified with sunflower proteins [80]. Enhancing the bars by adding other intense ingredients, such as cherries, coconut milk, and nuts, showed positive improvements in overall sensory acceptability, mostly due to the coconut’s strong flavor. In other words, the coconut flavor effectively masks the sunflower protein flavor. Hence, enhancing flavor must be taken into account when applying protein fortification. Another study used whey protein concentrate and vetch protein isolate. It indicated that at 4.35% and 6.00% concentrations, both proteins were significantly effective in increasing the protein content, with an excellent taste and overall sensory acceptability [95].

Date bars can also be fortified with different ingredients to boost their nutritional value, such as seeds, fruits, and plant extracts, or in more novel approaches, with food wastes, such as date pits. Other approaches can also be used, such as the germination or fermentation of the raw ingredients. In a recent study, Sukkari date bars were fortified with germinated flax seed powder in varied concentrations [96]. This approach significantly enhanced protein, fat, and mineral contents. Increased concentrations of seed powder led to a significant increase in protein content of 150%, in other words, 2.5 times higher than controls. Protein availability also showed significant enhancements based on higher contents of total amino acids recorded after seed fortification. Flax seeds are rich in protein content, ranging from 21 to 34% depending on genetics and environmental conditions. They are also rich in oils; approximately 41% of the seeds comprise fats [97,98]. Accordingly, fat content significantly increased by over 250% after adding 16% seed powder. Free fatty acid content was also enhanced; a decrease in saturated free fatty acids with increased monounsaturated and polyunsaturated free fatty acids was recorded. Flax seeds are also a good source of different minerals; increases in the presence of some minerals, such as phosphorous, magnesium, calcium, and sodium, were recorded. However, date bars containing the highest concentration of seeds (16%) were most disliked regarding taste, color, and overall acceptability, while concentrations ranging from 4 to 8% were accepted and scored a higher overall sensory acceptability. Adding more germinated flax seed content may necessitate enhancements in seasoning and flavor by adding other ingredients.

In another study, date bars were fortified with polyphenol extracts of pomegranate and apple peels [81]. This approach led to nonsignificant changes in the bars’ proximate composition, although it showed significant enhancements in the presence of total phenolics. The pomegranate peel extract showed the highest impact on increasing the total phenolics; at a concentration of 2%, it was equally effective to the apple peel extract at 3% due to lower amounts of polyphenols and flavonoids in the latter extract. Bars containing 2% pomegranate peel extract were also the most liked bars in terms of taste, flavor, texture, and overall sensory acceptability. Another study also explored the fortification of polyphenol extracts of moringa leaves or tamarind seeds at similar concentrations [99]. Moringa leaf extract at a concentration of 3% significantly increased total polyphenol contents by more than 900%. Accordingly, the radical scavenging activity of the fortified bars was significantly higher than that of bars with no added extracts. Regarding sensory acceptance, the most liked bars in terms of taste and overall sensory acceptability were those containing 2% moringa leaf extract, while bars containing tamarind were the most disliked. However, the two extracts differed by only one point.

The application of date fruit waste for preparing date bars was performed recently [100]; date pits were ground into powder and used to formulate date bars fortified with soy protein isolate. As a main protein source, fortification with soy protein led to a significant increase in protein content of 300%. However, adding date pit powder led to enhancements in protein content, recording an 8% increase. The fiber content also increased by 14% with adding date pit powder. The bars’ antioxidant potential also improved based on significant increases in radical scavenging activities of 33 and 65% for ascorbic acid and Trolox equivalents, respectively. However, the activity decreased during three months of storage, possibly due to exposure to higher temperatures.

In conclusion, the different formulations mentioned above indicate that preparing an excellent formulation of date bars could be challenging. Up to this point, the common formulations mainly focused on protein enrichment to produce balanced and protein-rich bars, as dates are not a main source of protein. Skim milk powder or its products, such as whey protein isolates or concentrates, were the most common protein sources used; as expected, significant increases in protein quantity and availability were achieved. Other protein sources such as cheese and plant-based proteins (e.g., sunflower or vetch protein isolates or concentrates) were also explored. However, the latter protein sources could significantly affect the bars’ sensory attributes. They must be applied under the studied concentrations, adding other taste-intense ingredients such as nuts or dried fruits. Furthermore, adding dry ingredients such as cereals or legumes can enhance protein quantity and availability. For instance, oat, wheat, barley, soybean, or chickpea flours can enhance the amino acid profile. Cereals can also contribute to significantly higher fiber content due to their richness in good-quality fiber such as β-glucan. In addition, relatively high amounts of minerals and vitamins can be achieved with cereal addition.

Cereal incorporation can not only promote significant enhancements in the nutritional attributes of the date bars but also result in significantly more enhanced texture and a more stable shelf life due to its ability to absorb moisture, leading to microbial growth limitation. On the other hand, incorporating other ingredients into date bars can not only target protein fortification or nutritional value in general; significant enhancements in the presence of bioactive or functional components can also be achieved. Functional ingredients such as extracts of fruits, plants, or seeds were shown to significantly increase phytochemicals such as phenolic acids or flavonoids, leading to a major increase in the antioxidant potential of the date bars. Although date fruit is considered rich in such bioactive components, which was one of the main reasons for its usage in developing energy bars, incorporating other nutritional ingredients can effectively boost dates’ antioxidant potential, providing an exceptionally functional product.

From another point of view, sensory attributes are key factors in the acceptance of any food product. Adding taste-intense ingredients such as nuts or dried fruits, besides their positive effects on nutritional value, can improve the date bars’ sensory acceptability regarding taste, flavor, odor, and color. Seasoning using different spices such as cardamom, cinnamon, or ginger and adding salt can strongly impact the taste and flavor. Apart from choosing between the different suitable and functional ingredients regarding sensory enhancements, processing techniques can also promote significant enhancements in the bars’ sensory attributes. Roasted ingredients such as cereals, dried fruits (mainly coconut flakes), and nuts significantly boost the sensory attributes regarding odor, taste, and flavor. However, applying high temperatures may affect the bioavailability of some micronutrients; therefore, such techniques should be performed with suitable periods and temperatures.

### 3.3. Potential Functional Effects of Date Bars

The high nutritional value of the prepared functional date bars presented in the previous section may play a strong part in promoting general health; in a recent pilot study, chronic kidney disease patients were given functional bars containing dates and other nourishing ingredients daily in combination with adherence to regular physical activity three times a week for three months; their measurements showed significant improvements in blood pressure levels, lipid metabolism, and other functional tests as compared to the control group, although those who consumed a functional bar daily with no weekly regular physical activity did not show such positive results, indicating the key impact of physical activity; however, the latter group had lower total cholesterol levels at baseline compared to other groups, indicating a possible explanation for the null results [101]. Nonetheless, the functional and pharmacological properties of dates have been discussed earlier. Therefore, date-based snack bars containing at least 60–70% date paste can be considered whole dates; hence, date-based snack bars could have a strong potential to promote different health-beneficial or functional effects. However, future studies regarding dietary interventions using date-based snack bars are suggested to produce more evidence and provide appropriate nutritional recommendations.

**Table 3:** Different formulations of date bars and their effects on proximate composition, mineral/vitamin content, sensory aspects, and other major outcomes.

Date Variety	Date Bars’ Ingredients	Proximate Composition (Average)	Minerals/Vitamins and Other Bioactive Attributes (Average)	Sensory Attributes	Reference
ND *	T0 (Control): Dates (100%) T1–T3: Dates (75%) Walnuts (15%) Single-cell protein (2.5%, 7.5%, or 5%) Dry skim milk (7.5%, 2.5%, or 5%) All bars (T0–T3) coated in milk chocolate	T1–T3 compared to T0: ↓ of 14–28% in moisture ↑ of 130–175% in protein ↑ of 300% in fat ↑ of 11–13.5% in energy	T1–T3 compared to T0: ↑ of 30% in Ca ↑ of 26% in Fe ↑ of 150–250% in P ↑ of 13–35% in Mg ↑ of 113–150% in B1 ↑ of 193–240% in B2	T1–T3 compared to T0: No significant changes. T1–T3 after 6 months of storage compared to initial time: T1–T3 stored at 20–37 °C (average): ↓ of 7% in flavor, texture, and overall acceptability. T1–T3 stored at 5 °C (average): ↑ of 8% in texture and by 5% in overall acceptability (indicating quality maintenance).	[77]
Ruzeiz	T0 (Control): Date paste (70 g) Almonds (11 g) Corn starch (19 g) T1–T3: Date paste (70 g) Almonds (11 g) Corn starch (7–11 g) Soy protein isolate (1.5–4.5 g) Dry skim milk (10.5–3.5 g) (T0–T4), half coated with milk chocolate, producing plain and chocolate bars	T1–T3 (plain, chocolate): ↓ of 10–15%, 5–10% in moisture ↑ of 27–83%, 16–38% in ash ↑ of 116–128%, 118–124% in protein ↑ of 6–16%, 36–42% in fat ↑ of 54–145%, 42–126% in fiber ↓ of 10–12%, 14–15% in NFE	T1–T3 (plain, chocolate) compared to T0: ↑ of 118–388%, 61–92% in Na ↑ of 10–56%, 1–26% in K ↑ of 57–138%, 33–92% in Ca ↑ of 48–95%, 44–68% in P ↑ of 12–29%, 4–17% in Mg ↓ of 32–2%, 26–4% in Fe	At initial (0 time): T1–T3 (average) compared to T0: ↓ of 1–4% in overall acceptability. Stored at 7 °C vs. 25 °C, 6 months: T1–T3 (average) compared to initial: ↓ of 9% vs. 7% in taste ↓ of 10% vs. 6% in texture ↓ of 9% vs. 8% in overall acceptability. T0 compared to initial: ↓ of 7% vs. 9% in taste ↓ of 7% vs. 16% in texture ↓ of 9% vs. 12% in overall acceptability.	[87]
Ruzeiz	T0 (Control): Date paste (70%) Almond (11%), Corn starch (19%) T1–T4: Date paste (70%) Almond (11%) Toruway (9–12%) or Zyest (2–4%) Dry skim milk (6.5–11.5%) (T0–T4), half coated with milk chocolate, producing plain and chocolate bars	T1–T4 (plain, chocolate) compared to T0: ↓ of 17–13%, 9–3% in moisture ↑ of 67–128%, 50–72% in ash ↑ of 72–117%, 76–110% in protein ↑ of 26%, 10–20% in fat ↑ of 9–68%, 16–74% in fiber ↓ of 13–8%, 11–8% in carbohydrates ↓ of 3–1%, 1% in energy	T1–T4 (plain, chocolate) compared to T0: ↑ of 26–61%, 23–44% in K ↑ of 133–141%, 69–85% in Ca ↑ of 15–23%, 9–21% in Mg ↑ of 192–265%, 91–125% in P ↓ of 36–11%, 32–20% in Fe	At initial (0 time): T1–T4 (average) compared to T0: No significant changes in taste, texture, or overall acceptability. Stored at 7 °C vs. 25 °C, 6 months: T1–T4 (average) compared to initial: No significant changes T0 compared to initial time: ↓ of 3% vs. 4% in overall acceptability.	[88]
Bushibal, Gash-Gaafar, Lulu, or Shahla	T0 (control): Date paste (70 g) Peeled almonds (11 g) Rolled oat flakes (19 g) T1–T3: Date paste (70 g) Peeled almonds (11 g) Rolled oat flakes (7–11 g) Skim milk powder (6–12 g) Sesame seeds (0–6 g)	T1–T3 compared to T0: ↑ of 47–51% in ash ↑ of 17–36% in protein ↓ of 66–36% in fat	T1–T3 compared to T0: ↑ of 31–46% in P ↑ of 104–117% in Ca ↓ of 15–10% in K ↓ of 37–18% in Fe ↑ of 33–12% in Zn	Comparing between four date varieties: Higher overall acceptability was noted in Lulu date bars, followed by Shahla (even after 6 months of storage).	[90]
Nabtat-Ali or Sukkari	Date pulp (500 g) Milk powder (100 g) Margarine (50 g) Citric acid (35.4 g) Pinch of table salt	Nabtat-Ali vs. Sukkari bars (%): Moisture (24.53 vs. 26.25) Ash (1.90 vs. 1.89) Protein (3.70 vs. 4.06) Fat (6.64 vs. 6.43) Fiber (5.51 vs. 4.6) Carbohydrates (57.72 vs. 56.77)	Nabtat-Ali vs. Sukkari bars compared to raw dates (%): ↓ of 27% vs. 17% in TPC ↓ of 39% vs. 29% in DPPH	Comparing the two varieties: ↑ of 9% in color scores of Sukkari bars, but overall acceptability was similar; both bars had less than 4 points (suggesting additional enhancement).	[91]
ND *	T0 (control): 100% date paste T1–T3: 80% date paste + (20% of either skim milk powder, soybean flour, or almond flour)	T1–T3 compared to T0: ↑ of 142–362% in protein (highest in skim milk bars) ↓ of 13–20 in fat, with ↑ of 494% noted in almond bars ↓ of 2–14% in carbohydrates ↑ of 109% in fiber, noted only in almond bars ↓ of 13–29 in vitamin C, with ↑ of 8% (only in soybean bars)	T1–T3 compared to T0: ↓ by 13–28% in vitamin C observed in bars containing almonds and skim milk, while bars containing soybean had ↑ of 8%.	T1–T3 (average) compared to T0: ↑ of 7, 2, 7, and 5% in flavor, aroma, color, and overall acceptability. Higher taste scores were recorded in almond bars. Higher color, flavor, texture, aroma, and overall acceptability scores were recorded for skim milk bars.	[79]
Aseel	Date paste (100 g) Soy and chickpea flours (12.5 g each) Oat (12.5 g) Skim milk (5 g)	Protein (1.95%) Fiber (3.30%) Fat (4.91%)	(ppm): Mg (412.9), Fe (20.9), Zn (19.7), Cu (3.34) and Mn (2.5)	Based on a 9-point hedonic scale: The sample received high scores of 7.6–8.5 in color, mouthfeel, and overall acceptability.	[83]
ND *	T0: Dates (100 g) Gram flour (5 g) Skim milk powder (5 g) Almonds and pistachios (5 g each) Cardamom and CMC (0.5 g each) T1–T3: T0 + Roasted oatmeal (15–35 g)	T2–T3 compared to T0: ↓ of 11–23% in moisture ↑ of 76–95% in ash ↑ of 25–47% in protein ↑ of 116–176% in fat ↑ of 63–94% in fiber ↑ of 11–18% in NFE ↓ of 6–9% in total sugars	T1–T3 compared to T0: ↓ of 74–75% in Fe ↑ of 62–162% in Mg ↑ of 52–75% in Zn ↑ of 142–193% in Mn	Based on a 9-point hedonic scale: Higher scores of 6.9–7 in flavor, taste, and overall acceptability were recorded for bars containing 25 g of oatmeal. Control bars received the lowest color, taste, texture, and overall acceptability scores.	[82]
ND *	Date paste (100 g) Roasted corn and gram flours (0–15 g) Skim milk powder (20 g) Almonds and peanuts (15 g each) Cardamom and salt (0.5 g each) Butylated hydroxyanisole (0.002%) Sodium benzoate and potassium sorbate (0.01% each)	(%): Protein (5.03–7.24) Fat (3.79–4.96) Fiber (1.74–2.22) Moisture (16.66–18.93) Ash (1.26–1.72) NFE (84.59–88.18)	ND	Based on a 9-point hedonic scale: Higher scores of 6.76–7.09 in color, flavor, and taste were recorded for corn bars.	[84]
ND *	T0 (control): Date (60 g) Date syrup and sesame seeds (20 g each) T1–T6: Date (30 g) Date syrup and sesame seeds (20 g each) Extruded wheat, necked barley, rice wheat (0–30 g each) Popcorn (0–30 g)	T1–T6 compared to T0: ↓ of 2–6% in moisture ↓ of 17–22% in ash ↑ of 16–33% in protein ↓ of 45–55% in fat ↓ of 1–85% in carbohydrates	T1–T6 compared to T0: ↓ of 4–15% in Ca ↓ of 3–8% in K ↑ of 3–29% in P ↓ of 12–27% in Mg ↓ of 9–24% in Fe ↑ of 8–35% in Zn	Based on a 9-point hedonic scale: Highest scores of 8.47–8.57 in color, taste, appearance, and overall acceptability for extruded wheat bars. The mixture of cereals scored lowest in all sensory attributes.	[21]
ND *	Date syrup: sesame paste (1:1, 2:1 and 1:2 ratios) Gelatin (7%)–TNLC (100–300 ppm) solution (25 g)	(g/100 g): Protein (6.12–10.81) and fat (15.75–31.5), highest in 1:2 ratio Carbohydrates (17.79–35.58), highest in 2:1 ratio	ND	Comparing different ratios: A ratio of 1:1 received the highest scores. Comparing different TNLC concentrations: 100 ppm received the highest scores in taste, texture, odor, and overall acceptability; 200 and 300 ppm were unacceptable due to thymol off-flavor.	[93]
ND *	T1–T4: Date paste (100 g) Dried apricot paste (15–30 g) Skim milk powder (25 g) Roasted gram (chickpea) flour (12 g) Roasted peanuts (25 g) Sodium chloride (table salt) (0.5 g)	T1-T4 (with increased apricot concentrations): ↑ of 12% in moisture ↑ of 3% in ash ↑ of 7% in protein ↑ of 0.3% in fat ↑ of 8.5% in fiber	T1-T4 (with increased apricot concentrations): ↑ of 0.7% in K ↓ of 1.5% in Ca ↑ of 5.2% in Fe ↓ of 37% in Mn ↑ of 2.6% in Zn ↑ of 17% in TPC ↑ of 35% in free fatty acids	Comparing apricot concentrations: A darker bar color was recorded with increased concentrations. Higher bar fracturability was recorded with increased concentrations.	[94]
Aseel	T1–T6: Dates (72–65 g) Dried apricots (8–17 g) Cheddar cheese (5–8 g) Whey protein isolate (12–13 g)	T1–T6 (%): Moisture (13.65–22.26) Protein (20.48–22.38) Fat (0.194–0.318)	↑ in TPC (with higher cheese concentrations). ↑ in TFC (with higher dates and apricot concentrations).	T1 (higher date concentrations): Received highest scores in taste. Bars with higher concentrations of whey protein isolate: Received highest scores in texture. T5–T6 (higher cheese content): Disliked due to savory and salty taste.	[4]
ND *	T1: Dates (64 g), dried apricots (16 g) Whey protein isolate (12 g) Cheddar cheese (8 g) T2–T3: T1 + roasted chickpea and rice flours (0–12.5 g)	T1–T3 (%): Moisture (22) Ash (2.12–2.44) Protein (23.2–236) Fiber (5.81–7.16) Fat (0.06–0.31)	ND	Bars containing 10% of roasted chickpea flour were the most accepted.	[78]
ND *	Date paste (1000 g) Roasted cashew nuts (170 g) Roasted hazelnut (150 g) Freeze-dried cherries (50 g) Inulin powder (50 g) Dry coconut milk powder (35 g) Dry coconut flour (25 g) Cinnamon (10 g), salt (5 g) Unpurified sunflower protein concentrate and isolate (varying %)	(%): Protein (8.9–15.9) Fat (12.8–13.6) Fiber (10.5–12.1)	ND	Comparing sunflower protein concentrations: All bars received high scores, but the highest scores in overall acceptability were recorded for control bars with 0%.	[80]
Karblain	Date paste (100 g) Roasted gram and corn flours (20 g) Peanuts and almonds (10 g each) Whey protein concentrate (varying %) Vetch protein isolate (varying %) Table salt (0.5 g), cardamom (1 g) Butylated hydroxytoluene (0.002%)	(%): Moisture (15.56–18.70) Ash (2.30–2.91) Protein (7.41–14.96) Fat (5.55–8.37) Fiber (3.58–3.91) NFE (70.85–81.12)	ND	Comparing between protein concentrations: Bars containing whey protein concentrate and vetch protein isolate at 6.05% and 4.35%, respectively, recorded the highest scores in taste.	[95]
Sukkari	T0 (control): Date paste (100%) T1–T4: Date paste (96–84%) Germinated flax seed powder (4–16%)	T1–T4 compared to T0: ↓ of 1–6% in moisture ↑ of 2–7% in ash ↑ of 49–151% in protein ↑ of 300–1300% in fat ↑ of 5–22% in fiber ↓ of 4–14% in carbohydrates	T1–T4 compared to T0: ↑ of 46–60% in Ca ↓ of 1–4% in K ↑ of 25–60% in Mg ↑ of 75–502% in Zn ↑ of 58–370% in Fe ↑ of 25–68% in Mn	Compared to T0: T1–T2 (4–8% of seeds): No significant difference was recorded. T3–T4 (12–16% of seeds): Mostly disliked in terms of appearance, taste, aroma, color, and overall acceptability.	[96]
ND *	T0 (control): Date paste (60%) Roasted gram flour (30%) Nuts (10%) T1–T4: T0 + pomegranate or apple peel polyphenol extracts (2–3%, in replacement of date paste)	No changes observed	Compared to T0: T1–T2 (2–3% of apple peel extract): ↑ of 400–600% in TPC T3–T4 (2–3% of pomegranate peel extract): ↑ of 500–800% in TPC	Bars containing 2% pomegranate extract received the highest scores in all sensory attributes, even higher than control bars.	[81]
ND *	T0 (control): Date paste (55%) Roasted gram and corn flours (16% each) Peanuts and almonds (6% each) Salt (0.9%), potassium sorbate (0.1%) T1–T4: T0 + moringa leaf polyphenolic or tamarind seeds extracts (2–3%)	(%): Total sugars (45.40–53.10)	Compared to T0: ↑ of 944% in TPC recorded in bars containing 3% of moringa extract. ↑ of 212% in DPPH recorded in bars containing 2% tamarind extract.	The highest scores in all sensory attributes were recorded for bars containing 2% moringa leaf extract, and the lowest scores were recorded for bars containing 3% tamarind seed extract.	[99]
ND *	T0: Date paste (100 g) Roasted corn flour (5 g) Cardamom (0.5 g) and salt T1–T3: T0 + soy protein isolates (16.44 g) or date pit powder (10.35 g) or both	T1–T3: ↓ of 12–34% in moisture ↑ of 1–5% in ash ↑ of 8–306% in protein ↑ of 21–52% in fat ↑ of 98–122% in fiber noted in date pit powder bars only, with ↓ of 11% in soy bars	Compared to T0: ↑ of 33–65% in antioxidant activity recorded for bars containing the highest date pit concentrations.	Bar color gradually decreased with an increase in date pit concentration.	[100]

Abbreviations: ND, Not Determined; ND * Not Determined (mentioned as date paste); T, Treatment; TSS, Total Soluble Solids; NFE, Nitrogen-Free Extract; TNLC, Thymol-loaded Nanostructured Lipid Carrier; CMC, Carboxy Methyl Cellulose; ppm, Parts Per Million; TPC, total phenolic content; TFC, total flavonoid content; DPPH, 2,2-diphenyl-1-picrylhydrazyl; Ca, calcium; Fe, iron; P, phosphorus; Mg, magnesium; B1, thiamine; B2, riboflavin; (↑), increase; (↓), decrease.

## 4. Conclusions and Future Work

This review summarizes studies showing the potential application of date fruit for developing highly nutritious snack bars. The main target of developing date-based bars was protein enrichment, as dates are high in carbohydrates and low in protein. Skim milk powder represented the most common and favorable protein source, with positive effects and no negative alterations in sensory attributes. Other protein sources, such as cheese and plant-based proteins, were also found to enhance the protein content but may negatively affect the sensory attributes. Their addition must be used under the studied concentrations while incorporating taste-intense ingredients such as nuts (e.g., almonds and walnuts) or dried fruits (e.g., coconut flakes).

Furthermore, protein enrichment is not the only target of developing date-based bars; providing an excellent functional snack bar can also be achieved. The reported studies have also used different nutritional and functional ingredients and showed significant enhancements in the presence of bioactive components, such as phenolics and flavonoids. However, the usage of the date fruit itself is promising in the snack bar industry due to its richness in various bioactive components. Dates’ bioactive components were demonstrated to promote several health-beneficial effects. Incorporating other nutritive ingredients besides dates can effectively boost dates’ functional properties. Among the different nutritious ingredients used in the reported studies in this review were extracts of fruit peels, plant seeds, or plant leaves. Such ingredients promoted significant enhancements in the bars’ functional and antioxidant potential. Moreover, the usage of date fruit wastes, such as date pits—seeds—was also shown to promote significant enhancements regarding nutritional and functional attributes.

This review can provide a background for future approaches to developing balanced, nutritious, and highly functional date-based bars with highly acceptable sensory attributes for consumers, including older adults, children, and athletes. Considering that the global snack bar market has shown remarkable growth trends, such products would be highly accepted, and thus, scaling up is strongly recommended.

## Figures and Tables

**Table 1:** Chemical composition of different date fruit varieties (range in %).

Date Varieties	Carbohydrates	Fiber	Lipids	Protein	Reference
10 date varieties	71–79		0.12–0.72	1.72–4.73	[6]
20 date varieties	47.8–81.4				[5]
12 date varieties		2.70–8.00	0.10–0.67	1.57–5.10	[7]
Sukkari	76.25	4.35	3.15	2.55	[26]
General Range	47–81	2–8	0.1–3.1	1–5	

## Data Availability

Data are contained within the article.
